# Continuing Professional Development ‐ Radiation Therapy

**DOI:** 10.1002/jmrs.758

**Published:** 2024-01-30

Maximise your CPD by reading the following selected article and answer the five questions. Please remember to self‐claim your CPD and retain your supporting evidence. Answers will be available via the QR code and online at www.asmirt.org/news-and-publications/jmrs, as well as published in JMRS — Volume 71, Issue 4 December 2024.

## Radiation Therapy — Original Article

### Situational anxiety in head and neck cancer: Rates, patterns and clinical management interventions in a regional cancer setting

Forbes E, Clover K, Oultram S, Wratten C, Kumar M, Tieu MT, Carter G, McCarter K, Britton B, Baker AL. (2024). *J Med Radiat Sci*. https://doi.org/10.1002/jmrs.736

1. In this study, what percentage of participants required anxiolytic medication at some stage during the treatment journey?
  - 2%
  - 5%
  - 10%
  - 12%
2. According to the pattern categories, what proportion of the sample were categorised as anxious (decreasing, increasing or stable anxiety)?
  - More than half
  - A quarter
  - A third
  - Two thirds
3. Which treatment session elicited clinically significant anxiety in the greatest percentage of participants?
  - Treatment planning (SIM)
  - Treatment 1
  - Treatment 2
  - Treatment 20
4. What is the main recommendation to ensure adequate support for patients experiencing persistent anxiety during the treatment journey?
  - Communication training for radiation therapy staff
  - Screening throughout the treatment journey
  - Provision of anxiolytic medication
  - More social support
5. According to this study, which screening tool is recommended to detect situational anxiety?
  - State Trait Anxiety Inventory
  - Distress Thermometer
  - Edmonton Symptom Assessment Scale
  - There is no established screening tool designed to assess situational anxiety.

### Recommended further reading

1. Forbes E, Clover K, Baker AL, Britton B, Carlson M, McCarter K. ‘Having the mask on didn't worry me until … they clamped my head down so I wouldn't move’: A qualitative study exploring anxiety in patients with head and neck cancer during radiation therapy. J Med Radiat Sci. 2023 Sep;70(3):283–291. doi: 10.1002/jmrs.658.
2. Nixon JL, Brown B, Pigott AE, et al. A prospective examination of mask anxiety during radiotherapy for head and neck cancer and patient perceptions of management strategies. J Med Radiat Sci. 2019 Sep;66(3):184–190. doi: 10.1002/jmrs.346.
3. Burns M, Campbell R, French S, et al. Trajectory of Anxiety Related to Radiation Therapy Mask Immobilization and Treatment Delivery in Head and Neck Cancer and Radiation Therapists' Ability to Detect This Anxiety. Adv Radiat Oncol. 2022 Apr 18;7(5):100967. doi: 10.1016/j.adro.2022.100967.

## Answers

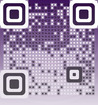

Scan this QR code to find the answers or visit www.asmirt.org/news-and-publications/jmrs.

